# Neuroendocrine Tumors: Challenges and Future Perspectives

**DOI:** 10.3390/jcm11154351

**Published:** 2022-07-27

**Authors:** Giuseppe Lamberti, Anna La Salvia

**Affiliations:** 1 Department of Specialized, Experimental and Diagnostic Medicine, Sant’Orsola-Malpighi Hospital, University of Bologna, 40138 Bologna, Italy; giuseppe.lamberti8@unibo.it; 2 Division of Medical Oncology 2, IRCCS Regina Elena National Cancer Institute, 00144 Rome, Italy

The awareness and interest of the scientific community towards Neuroendocrine tumors (NETs) has progressively increased in the last few decades. For instance, NETs have traditionally been considered rare tumors but their incidence has substantially increased over the last few decades, reaching 6.98 new cases/100.000 inhabitants per year in the latest 2012 update of the United States of America (USA) Surveillance Epidemiology and End Results (SEER) population registry [1,2,3]. Furthermore, NETs are overall considered indolent tumors, although their prognosis widely varies by tumor morphology and proliferation rate, primary tumor site, and stage.

Their heterogeneity in terms of biological aggressiveness, their variegated site of origin (even if they are most commonly gastro-entero-pancreatic in about 60% of cases and pulmonary in approximately 30% of cases), and their capability to potentially produce hormonally active substances pose unique challenges for clinical management. 

NETs are characterized by the peculiar and frequent expression on the cell surface of somatostatin receptors, which represents the ideal target for therapy (i.e., somatostatin analogues [SSAs] and peptide-receptor radionuclide therapy [PRRT]). Few chemotherapy schemes (such as streptozotocin/5-FU or the association of capecitabine and temozolomide) are currently part of the therapeutic armamentarium [4,5]. However, regardless of the large amounts of clinical trials and many promising new drugs, the only approved targeted agents for advanced progressive NETs are everolimus and sunitinib (the latter used only for those of pancreatic origin). Additionally, despite a great effort in the search for innovative biomarkers, only few validated biomarkers are available thus far. 

The current Special Issue, “Neuroendocrine Tumors: Challenges and Future Perspectives”, in the *Journal of Clinical Medicine*, is dedicated to collecting high-quality scientific contributions that mainly focus on recent advances in the context of the diagnosis, treatment, and the prediction of prognosis for NETs.

Currently, the increased use of endoscopy and imaging modalities of improved sensitivity have led to an increased detection of early-stage asymptomatic diseases [1].

Notably, functional imaging methods play a prominent role in NET diagnostics and therapeutic planning. In particular, somatostatin-receptor imaging (SRI) techniques such as scintigraphy (SRS) with 99m-technetium-octreotide or 111-Indium-pentetreotide (OctreoScan^®^, Mallinckrodt, Staines-upon-Thames, UK ) and positron emission tomography (PET) with 68-Gallium-labeled somatostatin analogues (for example, 68Ga-DOTATOC, 68Ga-DOTATATE, 68Ga-DOTANOC) are essential tools for the diagnosis and staging of well differentiated NETs [6]. These methods assess the expression of somatostatin receptors in neuroendocrine tumor cells, particularly of the subtype 2 (SSTR 2), and are thus also useful tools to predict responses to therapies targeting these receptors, such as *cold* SSAs and PRRT with radiolabelled somatostatin analogues [7]. PET with Gallium68-radiolabeled SSAs has replaced Octreoscan^®^ in many countries as it has greater sensitivity, specificity, rapidity, and spatial resolution, and enables the semi-quantitative estimation of metabolic activity by means of the standardized uptake value (SUV) parameter [8].

One study in the current Special Issue is focused on the role of 68-Gallium PET in type I gastric NETs [9]. Despite the fact that PET with Gallium68-labeled somatostatin analogues represents the standard of care in the diagnostic algorithm of all-sites NETs, given the indolent behavior in the majority of cases and the low risk of distant recurrence for type I gastric NETs, its role is not recommended for small lesions that are potentially suitable for endoscopic resection [10,11]. Nevertheless, rare cases of aggressive type I gastric NETs could be missed without staging using 68-Gallium PET. In this retrospective analysis of 15 patients, the 68-Gallium PET revealed itself to be particularly helpful for patients with a tumor size > 1 cm, in those cases where histological margins are not tumor-free after endoscopic resection (R1), or in grade 2 NETs. Indeed, functional imaging modified the management of patients with type I gastric NETs in 40% of cases (6/15). Notably, the authors encourage a discussion of each case in the NET-dedicated multidisciplinary tumor board and conclude that further prospective studies are needed to establish the role of 68-Gallium PET for in type I gastric NETs.

Two studies in the current Special Issue focused on NET treatment [12,13]. The first is a comprehensive review of the available literature and ongoing clinical trials of immune check point inhibitors (ICI) in well-differentiated lung neuroendocrine tumors, classified as either typical or atypical carcinoids [12]. In the immunotherapy and personalized therapy era, in which non-small cell lung cancer (NSCLC) and more recently also small cell lung cancer (SCLC) treatment strategies have been radically modified, only scarce data are available regarding the adoption of these agents in lung carcinoids. Overall, this work report details the preliminary efficacy data of this innovative therapeutic strategy for advanced and/or metastatic lung NETs. The most encouraging results have been reported with the combination of different ICIs, targeting CTLA-4 and PD-1. A synergistic mechanism has been suggested to explain this increased activity in comparison to single-agent ICI. Specifically, in the field of Lung NETs, the combinations of nivolumab plus ipilimumab and durvalumab plus tremelimumab have demonstrated to be particularly promising [14,15]. Currently, several studies are testing different ICI and ICI combinations together with chemotherapy and targeted agents. The results of these studies are eagerly awaited to establish the role of ICI therapy in lung NETs. Another work included in this Special Issue tackles the challenging issue of the treatment algorithm for rare neuroendocrine neoplasms, specifically pheochromocytomas and paragangliomas [13]. This review depicts a paradigmatic case, emphasizing the role of metoxycatecholamines assessment, which is inexpensive and non-invasive. Thus, the authors show how the metoxycatecholamines assessment could be useful for the early detection of disease recurrence or progression in this setting. This experience also highlights the value of the 68-Gallium PET for lesion detection, as recently reported in a systematic review and metanalysis of 31 studies [16]. However, the guidelines recommend TC and MRI as first steps in the diagnostic algorithm [17]. Finally, this work summarizes the available literature regarding genetics applications for these patients, to help clinicians towards a more personalized approach according to each case’s specific mutations.

In addition, one study in the current Special Issue is focused on NET-related quality of life [18]. Notably, the prevalence of these tumors has also significantly increased due to their indolent nature but also because of the improvements in therapeutic weapons and strategies for these patients [1,19]. Therefore, as survival improves, there is an increasing emphasis on optimizing the quality of life (QoL) among patients with NET, either under medical treatment or on active surveillance [20,21]. To date, only the European Organization for Research and Treatment of Cancer (EORTC) QLQ-G.I.NET21 questionnaire has been validated as a neuroendocrine neoplasm (NEN)-specific HR-QoL scale [22]. Indeed, the EORTC Quality-of-Life Questionnaire (EORTC QLQ-C30), which is frequently used in clinical practice, is a generic scale, addressing five functional domains (physical, role, emotional, cognitive, and social) in cancer patients [23]. However, it has been suggested that it is not adequate to cover specific NET-related symptoms and issues. Therefore, the main aim of this prospective, multicenter, international, and real-life pilot study was to collect patients’ opinions of these two scales through a dedicated survey. Overall, this analysis suggests that the validated scales have several limitations, preventing the correct evaluation of the side effects related to novel treatment agents. The approved questionnaires also have been suggested to lack in specific questions in relation to patients’ age and lifestyle, and the time points in the disease trajectory. 

In conclusion, the knowledge and the application of more proper diagnostic tools and specific instruments to assess patients’ QoL, as well as the continuous search for and development of new therapeutic strategies, are crucial for optimizing the treatment of patients with NET in the era of personalized medicine.

As the Guest Editors, we sincerely appreciate and would like to thank the reviewers for their insightful remarks and the JCM team’s support. Ultimately, we heartily thank all of the contributing authors for their valuable input.
